# Leading the way in pediatric sexual health screenings: evaluating pediatric emergency department workflows for the integration of STI screening tools

**DOI:** 10.3389/frhs.2025.1493318

**Published:** 2025-03-03

**Authors:** Laura Schubel, Deanna-Nicole Busog, Azade Tabaie, Monika Lemke, Danielle Foltz, Gia Badolato, Natasha Ajay Kaushal, Monika K. Goyal, Kristen Miller

**Affiliations:** ^1^ National Center for Human Factors in Healthcare, MedStar Health Research Institute, Washington, DC, United States; ^2^ Center for Biostatistics Informatics and Data Science, MedStar Health Research Institute, Washington, DC, United States; ^3^ Children’s National Hospital, Washington, DC, United States; ^4^ Center for Translational Research, Children’s National Hospital, Washington, DC, United States; ^5^ Emergency Medicine and Trauma Services, Children’s National Hospital, Washington, DC, United States; ^6^ Department of Pediatrics, George Washington University, Washington, DC, United States; ^7^ School of Medicine, Georgetown University, Washington, DC, United States

**Keywords:** emergency medicine, screening, sexual health, pediatrics, adolescents, workflow analysis, human factors

## Abstract

**Introduction:**

Emergency department (ED) encounters offer strategic opportunities for sexually transmitted infection (STI) screening, prevention, and treatment for adolescents at risk for STIs who may not otherwise have access to routine screening. This study determined optimal ED implementation of the Teen Health Screen (THS), a validated, tablet-based, patient-reported, sexual risk assessment, and evaluated its implementation feasibility under variable workflows and high-stress tasks.

**Methods:**

Workflow analysis included semi-structured interviews with patients, caregivers, and clinical staff members and clinical observations to understand patient and clinical workflow. The study was conducted in two urban pediatric EDs over six weeks. Participants included patients, parents/caregivers, registration staff, nurses, social workers, child life specialists, providers, and health IT experts.

**Results:**

The primary study outcome was development of a general model of ED workflow and patient-clinician engagement, focusing on patient flow, clinical tasks, people, and technologies involved. Workflow analyses identified key opportunities for THS deployment during the nurse assessment process, which aligns with other existing screening activities and offers privacy. This approach addresses potential barriers to integration such as privacy concerns, language and literacy barriers, the sensitivity of discussing sexual history, comfort with technology, tablet accessibility and security, and internet availability.

**Discussion:**

Workflow analysis provided valuable insights to the perceptions, thoughts, and practicality of implementing the THS in the ED. Interviews revealed general acceptance of the new process but highlighted logistical challenges, particularly with staffing and patient surge. Implementing the THS in ED settings appears feasible, with important opportunities identified for integration to improve patient safety, including staffing and workflow optimization.

## 1 Introduction

Adolescents and young adults account for half of all new sexually transmitted infections (STIs) annually (1–3), with racial and ethnic minoritized populations disproportionately affected due to inadequate access to healthcare and other socioeconomic factors (4). Many of these disparities can be attributed to unequal access to healthcare, including STI prevention and treatment services (4). The emergency department (ED) frequently serves as the primary or sole point of healthcare access for many adolescents, particularly those from marginalized or socioeconomically disadvantaged backgrounds (5–9). As such, the ED represents a crucial yet untapped resource for delivering sexual health services and integrating broadscale STI screening, prevention, and treatment strategies into broader public health efforts aimed at reaching underserved groups.

Despite the higher rates of STIs among adolescents who seek care in EDs compared to the general population (10–12), routine STI testing in these settings is uncommon, leading to significant under-detection and under-treatment of these infections (13–15). Research indicates that adolescents are interested in receiving sexual health education and services in the ED (16–18), however, they often feel uncomfortable disclosing sensitive health information during face-to face interactions with clinicians (19–24). The fast-paced, often chaotic nature of the ED environment, coupled with a lack of privacy, further complicates the provision of comprehensive sexual healthcare (25).

To address these barriers to STI identification and prevention in adolescents, we developed and validated the Teen Health Screen (THS), an electronic, patient-facing tool designed to identify adolescents at risk for STIs in the ED (26). The THS is a tablet-based screening that allows patients to privately and confidentially answer questions about their sexual health. Patient responses to the THS trigger an alert in the electronic health record (EHR) that enables providers to order STI or HIV testing in real time based on the patients’ request. The development process was guided by the unique challenges of the ED environment, including the need for efficient, non-disruptive tools that accommodate limited time and high patient volume. By supporting clinical decision-making, patient education, and rapid intervention, the THS leverages the ED's accessibility to provide timely STI testing, education, and treatment, thereby addressing critical unmet needs in adolescent sexual health.

Recognizing the importance of effectively integrating this new health information technology (IT) into the ED workflow, we conducted a comprehensive workflow analysis to identify optimal implementation strategies. This study describes the workflow analysis from qualitative interviews and clinical observations to ensure the successful implementation and uptake of the THS tool in real-world clinical settings.

## 2 Methods

### 2.1 Study design and setting

This study utilized a workflow analysis comprised of semi-structured interviews with patients, caregivers, and clinical staff members, and clinical observations to understand patient and clinician workflow in the ED. The provider interviews and patient observations were conducted at a Level 1 pediatric trauma center with two locations: a children's hospital with an annual ED volume of 90,000 visits, including approximately 19,000 adolescent (aged 15–21 years) visits, and a satellite ED within a community hospital in a historically under-served area with an annual volume of 37,000 visits, including about 6,000 adolescent visits. Research activities were conducted over six weeks from August to September 2022. All research activities were approved by our institution's IRB.

### 2.2 Data collection: semi-structured interviews

Semi-structured interview guides were collaboratively and iteratively developed by a multidisciplinary team, including experts in human factors engineering, health information technology, public health, clinical practice, and health services research. A sociotechnical systems approach informed the design of the guides, ensuring consideration of the complex interactions between people, technology, and processes in the ED environment (27). Five distinct interview guides were created for the following roles: (1) patients, (2) caregivers, (3) registration staff, (4) nursing staff, and (5) providers.

The interview guides included open-ended questions tailored to each role to explore participants’ experiences and perspectives on current health IT workflows, screening processes, and potential barriers to implementing a tablet-based STI screening process. Questions addressed topics such as the sequence and duration of ED workflows, interaction points with patients, use of technology (e.g., EHRs and mobile devices), and perceptions of existing patient-reported outcome data collection processes. For instance, patients and caregivers were asked about comfort with providing sensitive information via tablets, privacy concerns, and preferences for when and where such screening should occur. Clinical staff were queried on workflow integration, perceived challenges (e.g., staffing, time constraints), and logistical considerations such as tablet storage and management. General questions were included across all guides to examine participants’ views on the ED's role in preventative care, their engagement in promoting patient health, and perceived feasibility of implementing new screening workflows. Specific examples included exploring staff comfort with distributing and explaining tablet-based tools and patients’ preferences for accessible and private screening options. The comprehensive nature of the guides allowed for tailored yet comparative insights across all participant groups.

Recruitment of patients and caregivers occurred both on-site in the ED and via follow-up after a visit. For on-site recruitment, researchers were present in the ED to identify and recruit patient-caregiver dyads for interviews. Additionally, some interviews were conducted virtually using Microsoft Teams, either with the adolescent, the caregiver, or both, following their ED visit to accommodate participant preferences and scheduling constraints. Inclusion criteria included being between 15 and 21 years of age and presenting with a chief complaint unrelated to a sexual assault. Recruitment of clinical and hospital staff was conducted using snowball sampling to ensure a diverse range of perspectives. Clinical participants were purposively selected to capture variability in their backgrounds, roles, and hospital sites. Each interview lasted approximately 30 minutes and was conducted either in-person or virtually. On-site interviews were conducted during downtime or waiting periods for staff or patients/caregivers, respectfully in private areas of the ED. A minimum of two interviewers (DB, LS, AT, KM) conducted each session, with one serving as the primary interviewer and the other as a notetaker. All interviews were recorded and transcribed for subsequent data analysis.

### 2.3 Data collection: workflow observations

Clinical observations were conducted to capture detailed insights into ED workflows and interactions at both ED locations. These observations focused on the processes patients experience during an ED visit, interactions with clinical staff, and the use of tools and technologies in care delivery. Observing two distinct sites allowed for the evaluation of variations in patient volume, demographics, staffing, and geographic location.

The observations followed a structured guide designed to document key elements of the clinical workflow, including activities such as completing paperwork, receiving care, or undergoing diagnostic testing. Observers recorded individuals interacting with the patient, including caregivers, front desk staff, nurses, medical assistants, physicians, and others. The guide also captured information about tools and technologies used during the patient's visit, such as the EHR, tablets, paper forms, or other devices supporting clinical goals. Observers documented the physical location of each task (e.g., waiting room, exam room, procedural room) and contextual factors influencing the patient's experience, such as the availability of private spaces, crowdedness of the waiting area, or environmental distractions.

The observation guide was iteratively refined throughout the study to better capture emerging findings. For example, early observations highlighted critical contextual factors, such as challenges related to privacy, which informed subsequent adjustments to both the guide and the interview questions. Recognizing the need for private spaces during patient interactions led to the addition of targeted questions exploring the feasibility of conducting screenings discreetly within the ED environment. Similarly, findings on the roles of ED professionals informed the inclusion of questions about workflow integration and role responsibilities for administering the screening tool and providing follow-up care. This iterative approach ensured alignment with the study's objectives while addressing key barriers and facilitators identified during data collection.

All participants for the observational component of the study, including patients, caregivers, and clinical staff, were recruited on-site in the ED. Eligible patients were identified through the EHR and approached by observers in the waiting room. Inclusion criteria for patients included being between 15 and 21 years of age and presenting with a chief complaint unrelated to a sexual assault. Observers also engaged caregivers and clinical staff who met the inclusion criteria to ensure a comprehensive understanding of the observed interactions and workflows.

Each observation lasted approximately four hours, capturing multiple patient and clinician interactions and focusing on key roles such as registration, triage, and bedside care. To ensure consistency in data collection, two of four researchers (DB, LS, AT, KM) conducted the initial observations together. Subsequent observations involved one observer shadowing participants in key roles. Observers adhered to a non-intrusive approach to collect comprehensive data without disrupting clinical workflows.

Field notes documented clinical workflows, interactions between patients, clinicians, and caregivers, and the use of technologies. Observers recorded the flow of information, time spent on tasks, and contextual factors influencing care delivery. Aggregated data were used to develop workflow process maps and transition diagrams, detailing the sequence of interactions and the flow of information. These visual tools highlighted areas for potential improvement and informed the integration of the STI screening process within the ED workflow. This structured and iterative approach provided a comprehensive understanding of the patient journey and the practicalities of implementing new technologies in dynamic healthcare settings.

### 2.4 Data analysis

Data analysis followed an inductive approach, allowing themes to emerge directly from the data while being informed by the structure of the interview guides. Two researchers (AT, KM) jointly analyzed the initial set of interviews, engaging in open coding to break down the data into discrete excerpts that captured key ideas and patterns. These excerpts were categorized into initial coding categories aligned with the interview topics, such as workflow barriers, perceptions of technology, and preventative care in the ED. During this collaborative process, the researchers sat together to ensure consensus on coding categories and to develop a shared codebook. Once consensus was achieved, subsequent interviews were coded independently by the two researchers using the codebook, with regular check-ins to address any new codes or emerging themes (28).

To ensure accuracy and relevance, interviews were analyzed iteratively as they were conducted, rather than waiting until all interviews were complete. This iterative process allowed insights from earlier interviews to inform subsequent data collection by refining the interview guide to probe emerging themes more deeply. For example, early feedback on the use of technology for STI screening led to additional questions about patient comfort with privacy and data security.

Thematic analysis identified both overarching themes and group-specific subthemes. Broad themes included: (1) workflow barriers and facilitators, (2) patient and caregiver comfort with technology, (3) perceptions of ED staff roles in health promotion, and (4) challenges in implementing preventative care in the ED. These themes applied across interviews, but subthemes reflected the unique perspectives of patients, caregivers, and clinical staff. For instance, clinical staff often highlighted time constraints and competing priorities as workflow barriers, while patients emphasized the need for clarity and privacy during the screening process.

Observation data from the two EDs were analyzed separately by two researchers (DB, LS) who reviewed field notes collaboratively to draft and refine process maps for two ED groups. Observation notes were coded for workflow patterns and barriers, which informed the development of process maps using LucidChart (29), a web-based application for collaborative process diagramming. Once the initial maps were completed, the remaining observation data were reviewed independently, and process maps were iteratively updated. The process maps were combined into a single diagram to demonstrate overlap in processes and procedures, and differences between sites are further noted in the diagram. This combined approach of structured thematic analysis and inductive coding ensured the inclusion of both expected and emergent insights, providing a comprehensive understanding of participant perspectives across clinical groups and EDs.

## 3 Results

### 3.1 Participant demographics (interviews)

In total, 26 individuals were interviewed, though demographics for registration staff (*n* = 1), social workers (*n* = 1), and child life specialists (*n* = 1) are not reported in Table 1 due to small sample sizes. Table 1 includes demographics of *n* = 23 participants. The majority of participants self-identified as woman (*n* = 18, 78.3%). Among the patients interviewed (*n* = 5), most were 15–18 years (*n* = 4, 80.0%) and currently enrolled in high school (*n* = 4, 80.0%). Caregivers (*n* = 4) were primarily aged 40–50 years (*n* = 3, 75%) and held advanced degrees (*n* = 2, 50.0%). Clinical participants (*n* = 9) mostly reported being employed at their current institution and in their current roles for 0–5 years (64.3%).

**Table 1:** Demographics of patients, caregivers, and clinical interview participants.

Demographics	Patients (*n* = 5)	Caregivers (*n* = 4)
Gender		
Woman	5 (100%)	2 (50.0%)
Man	–	2 (50.0%)
Age (years)		
15–18	4 (80.0%)	–
19–21	1 (20.0%)	–
40–50	–	3 (75.0%)
50–60	–	1 (25.0%)
Highest level of education		
High school (current)	4 (80.0%)	–
High school degree or equivalent	1 (20.0%)	1 (25.0%)
College degree	–	1 (25.0%)
Advanced degree	–	2 (50.0%)
Demographics	Nurses (*n* = 3)	Providers (*n* = 11)
Gender		
Woman	2 (66.7%)	9 (81.8%)
Man	1 (33.3%)	2 (18.2%)
Overall length of employment		
0–5 years	1 (33.3%)	8 (72.7%)
6–10 years	2 (66.7%)	2 (18.2%)
10+ years	–	1 (9.1%)
Length of employment in current role		
0–5 years	1 (33.3%)	8 (72.7%)
6–10 years	2 (66.7%)	3 (27.3%)
10+ years	–	–
Specialized training		
Pediatric emergency medicine	–	5 (45.5%)
General pediatrics	–	2 (18.2%)
Other	–	4 (36.4%)

Demographics of registration, social work, and child life staff members are not included due to small sample sizes.

#### 3.1.1 Patient and caregiver perceptions of health screening in the ED

Patients and caregivers discussed their healthcare experiences over the prior year. Caregivers reported varied frequencies of healthcare use for their adolescents, with responses ranging from one visit to over 30 visits per year. Regarding ED visits, three patients reported visiting the ED twice in the past year. All patients recalled completing paper forms, often with caregiver assistance, and expressed a preference for electronic forms to reduce repetition and response burden. All caregivers agreed that the triage system of the ED was quick but wait times to see a provider were lengthy.

When asked about reporting sexual health information via electronic tablet-based survey, both patients and caregivers expressed comfort using an app or tablet, emphasizing the importance of privacy and the assurance that the information would be securely received by the care team. Caregivers indicated that it is very important for them to know that the information their teenager provides is received by the care team.

#### 3.1.2 Feedback on THS screening and implementation

Next, we aimed to understand when the most appropriate time would be to integrate the THS screening into the ED workflow (Table 2). Clinical staff described their job responsibilities and any related use of technology to provide insight into existing opportunities. Both registration staff and nurses described using the EHR for intake processes (e.g., registration staff recorded demographics, consent, etc., and nurses conducted initial assessments). When asked about utilization of the EHR, providers described activities like conducting physical exams, developing care plans, and recording diagnostic information. Providers also stated use of other electronically-integrated technologies, such as EHR-integrated cell phones, ultrasound machines, and tablet-based virtual interpreters. Common screenings currently used in the EDs included suicide risk and asthma. These were typically conducted by a nurse during triage, though screenings would vary or be added based on patient age and reason for visit.

**Table 2:** Identified challenges to implementation for screening, including the interview groups that cited the concern and relevant quotes.

Challenges to screening implementation	Interview groups	Exemplary quote(s)
Lack of privacy and appropriate space	Providers, child life, caregivers	“With the ED, the way it is right now, there's just not a lot of… patients that get rooms or private space, especially for low acuity complaints.”—Provider 5
Parental/caregiver influences	Providers, registration, social work, nurses, patients	"Are you really going to get the truth? If Mom is over the shoulder, I doubt it.”—Registration 1
Technology literacy	Providers, nurses	“…And the other thing… is just technology itself. I mean, it depends on the comfort of the person filling it out. I think that people are quick to give up if… they run into problems.”—Provider 1
Subjective patient comfort with the topic	Providers, child life, social work	“…I think there is going to be some people who just feel uncomfortable answering some of those questions. But I think that should be probably a minority because in my opinion… I feel like kids these age… are so used to interfacing with a tablet and actually feel more uncomfortable with a person than they do with the tablet.”—Provider 1
Language and literacy barriers	Nurses	“We do get a fair amount of Spanish speaking patients, so [it's important] they can easily flip over to Spanish or another language.”—Nurse 2
Workflow/staffing concerns	Providers, nurses	“Having adequate staffing would be really helpful… I [worked] two days ago and our emergency room only holds 38 patients and we had 60 patients across the board. So, there are patients in the hallway, in every hallway. And I think that having the shortage of nurses that we did that day also was detrimental to patient care because we couldn't be everywhere at once and take care of every single patient to the best of our ability.”—Nurse 3
Distribution and ownership of the tablets	Providers, nurses, caregivers	“[Deciding who should distribute tablets] is tough because I feel like everybody is spread so thin.”—Provider 2
Tablet security	Providers, nurses, social work	“[You should make] sure that the tablets don't work outside the hospital, and make sure that they're labeled so people don't think like ‘oh, free [tablet].”—Nurse 1
Internet availability in the ED	Providers, social work	“[Our team] really wanted to do a screening where we were able to push these screens directly to families', or parents', caregivers', phones… but unfortunately there's just so many connectivity issues in the ER… like half the time people wouldn't even receive the links to their phone or and their e-mail. And we ended up spending a lot of time being like IT support for families…”—Social Work 1
Clinical time to review and discuss results	Providers	“I definitely do [have concerns about the time to review]… it's just an incredibly busy environment in the ED… you're constantly triaging information… [Reviewing] is like the 20th thing that I need to address right now.”—Provider 9

Clinical participants and health IT experts provided varied suggestions for integrating the screening into the ED workflow, ranging from use in the waiting room to during triage, with a consensus that it should occur early in the patient visit. When asked about where the screening should occur, patients and caregivers expressed concerns about privacy and felt most comfortable answering questions in a private room as compared to the waiting area (e.g., registration). This was contrary to some clinical participants’ opinions. As one provider noted, “[The waiting room] is the best time to capture that information because, essentially, there's nothing happening during that time period.” Participants noted that using tablets would expedite the screening process, facilitating faster care processes and allow for direct transfer of information into the EHR.

Participant solutions included reviewing the screening tool to ensure an appropriate reading level, asking brief and concise questions to expedite completion, ensuring patients understand the screening is voluntary, including resource information for teenagers with a history of sexual trauma, labeling the tablets with the hospital's information and clear instructions on where to return once the screening is completed, adding the screening as an EHR requirement based on patient age, returning all tablets to a “dirty bin” for sanitization, using location trackers or other means of finding missing tablets, sharing the screening results with health providers in a way that is easy to interpret, and ensuring providers’ awareness of a completed screener.

Several interviewees also brought up the topic of cost with assumed work. As stated by the registration staff member: “If you're gonna give me an FTE (full-time employee) we could do the survey all day, right?.. It is more than just a tablet, you gotta have someone who's going to absorb the work.” This highlights not only the burden of additional workload, but potentially the costs associated with staffing changes that may be needed to effectively implement this work. Similarly, a physician suggested switching to smartphones instead of tablets as a way of improving the workflow and possibly being more cost-effective: “…QR codes could actually work really well, because I think most people [have] a smartphone. We would just need to buy a bunch of chargers.”

#### 3.1.3 Preventative care in the ED

Prior to ending interviews, clinical participants were asked for their opinions on providing preventive care services in the ED. The majority of providers and nurses agreed that the ED should offer preventive care, where possible. As stated by one provider, “I feel like sometimes the preventive care that you provide in the emergency department is some of the best work we do, because it's usually [provided to] the most vulnerable patients [since] they're not getting it anywhere else.” However, several participants disagreed and felt more hesitant towards the prospect. One provider noted: “We are there for emergency care, and while it's certainly great to catch people before anything bad happens, I don't want us to overstep the role of what the emergency physician should be doing.. The more that we offer these screening and preventive functions, then with the more we are at risk of making family physicians and general practitioners less important…”

### 3.2 Findings from workflow observations

**Figure 1:**
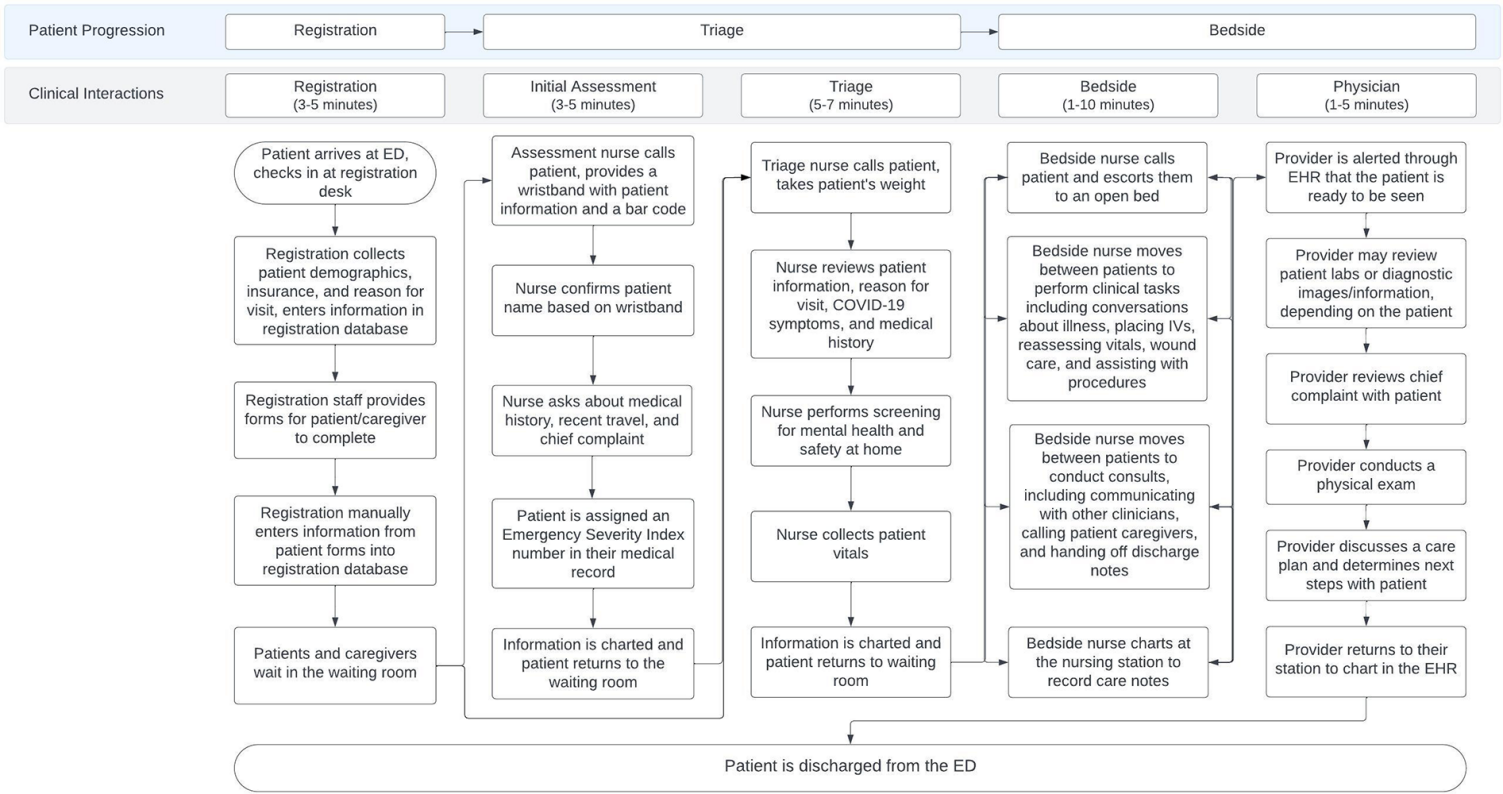
Workflow map of the typical patient progression through the ED based on clinical observations with registration staff, triage nurses, bedside nurses, and providers. Time ranges represent the shortest and longest observation at each point in the patient progression. The two step triage process was only present at location A.

A total of 20 observations were completed in two ED locations, providing insights into the patient journey through the ED, highlighting three major points of progression: registration, triage assessment, and bedside assessment (Table 3). Between each progression point, documented waiting times varied. Four or five primarily clinical or staffing interactions occurred with the patient throughout their journey depending on the location, including registration staff, assessment (location A), triage, bedside nurses, and providers. Variations in workflow were influenced by location, workflow responsibilities, staffing levels, patient volume, and the specifics of patient presentation including patient acuity. Each point of progression and each clinical interaction included separate workflows and occasionally varied staff members (Figure 1).

**Table 3:** Professional roles of observed participants across two ED settings.

Professional role	Location A	Location B
Patients and caregivers	2	2
Providers	1	2
Nursing (bedside)	2	1
Nursing (triage/assessment)	2	2
Nursing (pivot)	2	0
Registration staff	2	1
Nursing (fast track)	1	N/A

Significant differences were noted between the two hospitals sites. Location A, a higher-volume children's hospital, utilized a “fast track” system for lower-acuity patients and a two-step triage process to streamline care. In contrast, workflows at location B were described as a quicker and more seamless progression through the clinical care setting. This can be attributed to patients of lower acuity and may require fewer intensive resources and/or care. The two-step triage process includes an additional assessment where a nurse conducts a high-level verbal assessment of the patient immediately after the patient registered with the front desk. This two-step occurred separate to the triage process and was leveraged as a way of streamlining clinical services for patients of higher acuity.

Among staffing observations, no differences emerged with registration between locations. The workflows of nurses in triage were highly similar across both institutions in the initial screenings and data collected, though differences emerged dependent on the patient's presenting illness and acuity. For example, medications may be supplied to a patient presenting with a fever, and different questions would be asked to a patient presenting with a physical concern compared to a mental health concern.

Bedside nurses’ workflows were highly variable depending on the patient's clinical needs (e.g., medication administration, vitals assessment) and administrative work (e.g., obtaining information, prior authorization). Variations included tasks specific to patient care and what was necessitated, rooming patients, charting, room turnover, minor procedures, placing IVs, administering medication, collecting patient information, coordinating with providers, providing meals, transporting patients, communicating with caregivers, and providing discharge instructions. Responsibilities varied depending on the patient, staffing, time of day, and point in the patient's journey.

Provider workflows, similar to bedside nurses, were highly variable depending on the patient, time of day, and location. Variations in physician workflows included discussing the patient with other health professionals (resident to attending communications, consultations either provided or received, discussing care plans with nurses etc.), and occasionally conducting discharge work depending on the patient and the hospital site.

These observations complemented interview findings by providing a snapshot of the challenges described by participants. For instance, interviewees highlighted privacy concerns with conducting the screening in open spaces like the waiting room. Patient observations corroborated these challenges, noting the limited availability of private spaces, particularly for low-acuity patients. Similarly, while caregivers and clinical staff expressed preferences for leveraging existing workflows during registration or triage for implementing the screening, observations revealed the variability in workflows and staff responsibilities that could complicate consistent implementation. This alignment between interview and observational data underscores the importance of tailoring the integration of the tool to the unique workflows and spatial constraints of each ED.

Additionally, observations provided insights into the roles and responsibilities of ED staff that informed the feasibility of assigning tasks related to the screening tool. For example, the observed variability in bedside nurses’ and providers’ workflows highlighted potential barriers to assigning additional responsibilities, aligning with interview findings that cited staffing and workflow concerns as challenges to implementation. Observations also revealed potential strategies for mitigating these challenges, such as embedding the tool into existing EHR workflows or leveraging fast-track processes for low-acuity patients.

Overall, combining interviews and observation data allows for a richer understanding of the logistical challenges and opportunities for integrating the THS tool into the ED environment. These findings demonstrate the need for site-specific adaptations to address workflow variability, spatial constraints, and stakeholder concerns while leveraging existing strengths, such as familiarity of staff with the EHR-integrated processes.

## 4 Discussion

The analysis of semi-structured interviews and clinical workflows provided important insights into the feasibility and practicality of implementing the THS in the ED. While there was general acceptance of the process and perceived value in improving patient safety, participants voiced concerns about logistical challenges, particularly related to staffing and patient surges. Observations further revealed the variability in workflows across both EDs, highlighting that care processes differ significantly based on staffing, patient needs, and hospital-specific procedures.

A major finding from this study was the variability in clinical processes, especially in relation to the initial assessment process and wait times. Clinical processes are highly variable both among and between staff members and hospitals (30–32). This variability underscores the need for flexibility in implementing interventions like the THS. ED environments are inherently dynamic, shaped by fluctuating patient volumes, diverse presenting concerns, and staffing patterns that can shift from one shift to the next. Hospitals employed different triage systems, such as fast-tracking low-acuity patients or conducting additional assessments upon arrival, which further complicates a one-size-fits-all approach to workflow integration. Such inconsistencies suggest that any tool designed for ED use must be adaptable, aligning with both high-acuity and routine workflows while minimizing disruption to the existing care delivery model. Future implementation efforts should account for the idiosyncrasies of each hospital and consider customizing deployment strategies based on institutional differences.

Time constraints emerged as a critical factor influencing the successful implementation of the THS. Clinical staff, particularly nurses and providers, expressed concerns about fitting a screening tool into an already compressed clinical timeline, particularly in the context of ED overcrowding—a persistent issue exacerbated by factors like seasonal fluctuations in patient volume and the aftermath of the COVID-19 pandemic. While individual interactions were typically brief, the cumulative effect of ED overcrowding and patient throughput requires thoughtful consideration of where and how to incorporate the THS. As observed, the clinical processes between wait times are quick but productive, with average times of less than ten minutes across all interactions. Especially in the era of ED overcrowding (33–36), the time allocated for screening necessitates careful consideration of who is asked to store, charge, sanitize, introduce, and administer the screening, along with the response burden on the respondent. Screening therefore must be adapted to meet the needs of the clinical environment to increase adherence and acceptability (37–40). The analysis identified downtime between key points in the patient journey, such as the intervals between registration and triage or triage and bedside assessment, as opportune moments to introduce the screening. Conducting the THS early in the process would ensure that its results are available before more time-sensitive clinical decisions are made, streamlining the care pathway while potentially reducing overall ED wait times. In this way, the THS could complement, rather than compete with, the clinical workflow, allowing for more efficient care coordination.

Privacy remains a major concern when conducting sensitive screenings in the ED, particularly in an environment that lacks private spaces and in the context of sexual health screening, where adolescents may be reluctant to disclose sensitive information in a public or semi-public environment. This aligns with studies that commonly cite privacy as a barrier to screening in the ED which is often open with a chaotic layout (25, 41). The findings from this work suggest the need for privacy considerations when conducting screening, including privacy from caregivers to not influence responses, from other patients, and to disclose questions, concerns, or potential trauma that may be triggered from the screening. Electronic screening therefore allows for the provision of confidential screening in a busy work environment with non-private setting, and careful consideration of the material and language used in the screening is necessary to ensure patient emotional safety. The THS benefits from tool validation and prior work to ensure acceptability to adolescents in the ED, especially among those who declare sexual experience (12, 42–44).

The study also revealed broader concerns related to logistical and operational challenges in deploying an electronic screening tool in the ED. Participants raised issues such as the availability and management of tablets, ensuring reliable internet access, and the additional responsibility placed on clinical staff to introduce, monitor, sanitize, and charge the devices. These considerations highlight the importance of not only having the right technology in place but also having clear protocols and adequate staffing to manage the process effectively. Solutions proposed by participants, such as using location trackers for tablets or incorporating screening into the EHR as a mandatory task, point to ways in which these challenges can be addressed. Ensuring buy-in from clinical staff is another critical factor, as the success of the THS will depend on its seamless integration into their already demanding workflows.

This study has limitations. The findings are drawn from two pediatric EDs in the US which may limit generalizability to other settings or populations. The opinions of an acceptance of a screening for sexual health may vary by region, culture, and beliefs. Additionally, there is a potential for selection bias, as participants who consented to the interviews may have been more open to the topic than others who declined participation. Furthermore, all the patient participants interviewed in this work identified as women, potentially limiting generalizability. Future research should explore the implementation of the THS in diverse settings and with different populations to build a more comprehensive understanding of its feasibility and effectiveness.

## 5 Conclusion

This study highlights the potential of the THS tool to transform the identification and management of STI risks among adolescents in the ED setting. By leveraging a confidential, patient-friendly, and efficient electronic platform, the THS tool addresses critical barriers in sexual health communication and empowers adolescents to share sensitive health information. The integration with the EHR enables seamless data sharing, potentially enhancing clinical decision-making and supporting timely testing, education, and treatment interventions.

At the same time, our findings reveal significant challenges that warrant further attention to ensure successful implementation. Issues such as privacy concerns, workflow disruptions, and role delineation among ED staff emerged as key barriers to adoption. Addressing these challenges will require targeted strategies, such as optimizing physical spaces for privacy, refining workflows to minimize disruptions, and engaging all stakeholders—clinicians, caregivers, and patients—in the design and adaptation process. These insights emphasize the importance of balancing technical innovations with the realities of the clinical environment.

Despite these challenges, the THS tool demonstrates promise in advancing adolescent sexual health by fostering confidential, equitable, and patient-centered care in the ED. Future work should focus on iterative development and rigorous evaluation of the tool in diverse clinical settings to address identified barriers and ensure it meets the needs of both patients and providers. Ultimately, this study underscores the importance of designing and implementing health IT tools that align with the complex dynamics of healthcare delivery while supporting patient and provider goals.

## Data Availability

The raw data supporting the conclusions of this article will be made available by the authors, without undue reservation.
